# Structural Analysis of Mitochondria in Cardiomyocytes: Insights into Bioenergetics and Membrane Remodeling

**DOI:** 10.3390/cimb45070385

**Published:** 2023-07-21

**Authors:** Raquel A. Adams, Zheng Liu, Chongere Hsieh, Michael Marko, W. Jonathan Lederer, M. Saleet Jafri, Carmen Mannella

**Affiliations:** 1 Krasnow Institute for Advanced Study and School of Systems Biology, George Mason University, Fairfax, VA 22030, USA; radams12@gmu.edu; 2 Wadsworth Center, New York State Department of Health, Albany, NY 12201, USAmike.marko.em@gmail.com (M.M.); 3 Department of Physiology, School of Medicine, University of Maryland, Baltimore, MD 21201, USA; jlederer@som.umaryland.edu; 4 Center for Biomedical Engineering and Technology, School of Medicine, University of Maryland, Baltimore, MD 21201, USA

**Keywords:** mitochondria, cristae, cardiomyocytes, myofibrils, membrane remodeling, electron microscopy, electron tomography

## Abstract

Mitochondria in mammalian cardiomyocytes display considerable structural heterogeneity, the significance of which is not currently understood. We use electron microscopic tomography to analyze a dataset of 68 mitochondrial subvolumes to look for correlations among mitochondrial size and shape, crista morphology and membrane density, and organelle location within rat cardiac myocytes. A tomographic analysis guided the definition of four classes of crista morphology: lamellar, tubular, mixed and transitional, the last associated with remodeling between lamellar and tubular cristae. Correlations include an apparent bias for mitochondria with lamellar cristae to be located in the regions between myofibrils and a two-fold larger crista membrane density in mitochondria with lamellar cristae relative to mitochondria with tubular cristae. The examination of individual cristae inside mitochondria reveals local variations in crista topology, such as extent of branching, alignment of fenestrations and progressive changes in membrane morphology and packing density. The findings suggest both a rationale for the interfibrillar location of lamellar mitochondria and a pathway for crista remodeling from lamellar to tubular morphology.

## 1. Introduction

Mitochondrial bioenergetic output in the form of ATP is maximized by infolding the energy-transducing inner membrane (IM). Increasing the surface area of the IM packs more chemiosmotic machinery per mitochondrial volume into eukaryotic cells, freeing up space for the specialized functions that make eukaryotic life interesting [1]. However, this strategy for increasing ATP output carries both opportunities and risks [2]. The IM infoldings, called *cristae*, are invaginations on the periphery of the membrane (the inner boundary membrane, IBM) generated at narrow tubular necks or junctions [3,4,5]. There is growing experimental and computational evidence that these crista junctions (CJs) restrict the diffusion of ions, metabolites and soluble proteins, creating crista micro-compartments that are functionally distinct from the peripheral space between the IBM and outer membrane, OM [6,7,8,9,10,11,12,13]. The junctions are formed by a large protein–cardiolipin complex, MICOS (mitochondrial contact site and cristae organizing system), that has its origins in ancestral alpha-proteobacteria [14] and interacts with respiratory complexes, F_1_F_0_-ATP synthase dimers and lipids [15,16,17,18]. Recent studies suggest that crista junctions are dynamic structures that, like the cristae themselves, are continuously remodeled depending on the metabolic state [19,20]. A recent computational study indicates that metabolic steady states inside cristae can vary considerably with crista size and shape [13]. Increasing the crista length slows the return of ADP into the matrix, which in turn can decrease the flux of ATP synthesis. Since cells and tissues with high energy needs tend to have larger and more densely packed cristae, the mitochondrial design must optimize internal diffusion (for energy metabolism) while dealing with the physical stresses associated with cellular activities, such as transport along the cytoskeleton in neuronal cells and contraction cycles in the muscle. This paper explores how this is achieved in cardiac muscle mitochondria, using observations from transmission electron microscopic (TEM) tomography.

Mitochondria occupy around one-third of the volume of cardiac myocytes (e.g., [21]), presumably the upper limit that still allows sufficient space for contraction machinery and essential organelles. There are distinct subpopulations of mitochondria within cardiomyocytes that vary in terms of location and structure. Some mitochondria reside in ordered rows sandwiched between myofibrils, others occur singly or in clusters, proximal or distal to myofibrils, some abutting the sarcolemma and others the nuclear envelope, e.g., [22,23]. It is thought that the orderly arrangement of mitochondria between myofibrils provides an energetic advantage for the muscle [24], although the location may present challenges in terms of mechanical stresses [25]. Besides variation in cellular locale, cardiac mitochondria have considerable heterogeneity in interior design, falling broadly into two classes, those with extended flat lamellar cristae and those with tubular cristae. There is considerable disagreement in the literature about whether variable crista morphology reflects functional differences in mitochondria at different locations within myocytes [22,26,27,28]. This issue provides a major focus for the current report, which is a survey of the topology of the inner membranes of mitochondria in normal adult mammalian (rat) ventricular cardiomyocytes using electron tomography. We devise a classification for crista morphology based on a dataset of 68 mitochondrial subvolumes in 58 tomograms, and determine significant correlations within the dataset between mitochondrial structure and cellular location. We discuss the functional implications of these correlations in terms of the influence of crista morphology on mitochondrial ATP output and response to physical stress. Finally, we suggest a pathway for the transition from lamellar to tubular cristae based on the close examination of cristae within individual mitochondria.

## 2. Materials and Methods

### 2.1. Preparation of Rat Ventricular Myocytes for Transmission Electron Microscopy

Isolated ventricular myocytes were obtained from adult male Sprague–Dawley rats using previously described procedures [29]. Hearts were excised from animals after intraperitoneal heparin injection and isoflurane inhalation, quickly immersed in an ice-cold isolation buffer (130 mM NaCl, 5.4 mM KCl, 0.5 mM MgCl_2_, 0.33 mM NaH_2_PO_4_, 10 mM D-glucose, 10 mM taurine, 25 mM HEPES and 0.5 mM EGTA; pH adjusted to 7.4 with NaOH), then perfused for 5 min on a Langendorff apparatus with an isolation buffer at 37°. Perfusion was switched for 6–8 min to the EGTA-free isolation buffer containing a protease cocktail. The heart was dismounted, dissected and the ventricles transferred to an isolation buffer containing 2 mg/mL BSA and 20 mM 2,3-butanedione monoxime, and was then rapidly minced, disrupted and filtered through a nylon mesh (pore size 300 μm). The myocyte suspension was allowed to settle and was prepared for TEM according to the protocol in [30]. Myocytes were fixed by resuspension in 2.5% glutaraldehyde in 0.1 M cacodylate (pH 7.2) and transferred to the Microscope Facility of the Johns Hopkins School of Medicine (Baltimore, MD, USA), where the sample was postfixed with 2% osmium tetroxide in a cacodylate buffer, dehydrated and embedded in Epon 812.

### 2.2. Electron Tomography and Structural Analyses

Epon blocks of rat ventricular myocytes were transferred to the Advanced Electron Microscopy Group at the Wadsworth Center (New York State Department of Health, Albany, NY, USA) for electron tomographic data collection. Sections were cut by ultramicrotomy to nominal thicknesses of 250–400 nm, and placed on the center of 200-mesh copper grids, pre-coated with a 50 nm Formvar film. To promote even penetration into the thick sections, uranyl acetate and lead citrate stains were applied to both sides for times that were thickness dependent. For uranyl acetate (which penetrates slowly), a saturated solution was applied at 60 °C for 15 to 30 min, while lead citrate was applied at room temperature for 6 to 10 min. After staining, a 5 nm carbon film was evaporated on one side of the grids to improve section stability during data collection, and 20 nm colloidal gold particles were deposited on the grids as fiducial markers for image alignment.

The tomographic tilt series were recorded using a JEOL JEM-4000FX equipped with a Gatan GIF2002 energy filter. The microscope was operated at a 400 kV acceleration voltage with zero-loss energy filtering using a 20-eV slit. The single-axis tilt series were collected at a 1° increment, from +60° to −60°, using a CCD camera with a 1024 × 1024 detector array (pixel size 1.8 or 1.6 nm). Tomograms were computed by weighted back-projection [31] and denoised by anisotropic diffusion [32] using IMOD (version 34.12.25) [33] and SPIDER (version 5mp.106) [34] software.

For visualization and quantitative analyses in Section 3.1, Section 3.2, Section 3.3 and Section 3.4, rolling averages of X–Y slices (i.e., slices from tomograms parallel to the section plane and normal to the electron beam at 0° specimen tilt) were computed from every three adjacent slices in the tomograms. Membrane lengths were measured by manual tracing of tomographic slices using tools in IMOD, which were also used to render the 3-D surfaces. Mitochondrial cross-section parameters were measured from displayed tomographic slices using grid overlays. Segmentation and quantitation in Section 3.5 was done using a novel, semi-automated protocol [35]. Following denoising by anisotropic diffusion, Frangi filtration [36] was applied in Microscopy Image Browser (version 2.83) [37] to enhance detection of vessel-like surfaces. Segmentation was performed by highlighting a few regions of interest (such as groups of cristae) for thresholding and extracting a polygonal mesh from the isosurface with the marching cubes algorithm in MATLAB (version 9.12 R2022a). Blender (version 2.93.1) was used for surface rendering and triangulation, and BlendGAMer (version 2.0.7) for measuring surface areas and volumes.

## 3. Results

### 3.1. Classification of Cardiomyocyte Mitochondria

Electron tomograms were obtained of mitochondria in 58 regions of normal adult rat cardiac muscle, using specimen preparative, image recording and processing protocols as described in Methods. The tomographically reconstructed volumes are slabs of 1.8 × 1.8 or 1.6 × 1.6 μm^2^ in area and 0.25 or 0.37 μm thick, containing only a part of the individual mitochondria which can exceed 1 μm in length. Regions were selected by criteria and were intended to provide a diverse sampling of mitochondria in terms of crista morphology and cell location. Thus, each tomogram contains one or a few mitochondria with easily recognizable cristae (broadly lamellar or tubular) that may be located in the vicinity of myofibrils (“interfibrillar”) or the plasma membrane (“subsarcolemmal”), in some cases within closely packed clusters. Since selection was not strictly random, the dataset does not provide information about the frequency of occurrence within cardiomyocytes of mitochondria with a particular size, crista morphology or cell location, and global averages of structural parameters in the dataset are not reliable. However, no attempt was made to select mitochondria with a particular size or crista morphology at specific locations inside the cells. Therefore, statistically significant correlations among mitochondrial size, crista morphology and location within the dataset should be meaningful.

**Figure 1:**
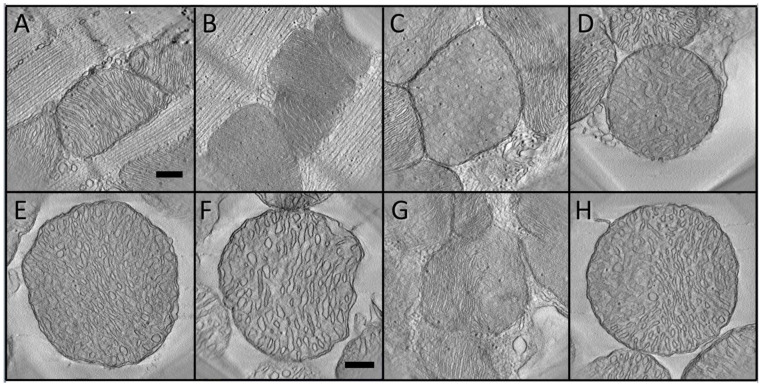
Central slices from tomograms representing the four classes of crista morphology in the dataset, as described in text. (**A**,**B**) Lamellar cristae in orthodox (**A**) and condensed (**B**) conformations. (**C**,**D**) Tubular cristae in predominantly transverse (**C**) and longitudinal (**D**) views. (**E**,**F**) Transitional crista morphologies. (**G**,**H**) Mixed cristae with about equal fractions of lamellar—tubular (**G**) and tubular—transitional (**H**) morphologies. Scale bars in (**A**,**F**) represent 250 nm. The scale bar in (**A**) also applies to tomographic slices (**B**−**E**,**G**,**H**).

The 58 tomographic volumes sample 68 mitochondria that could be grouped using the information in the tomograms into four distinct classes of crista morphology: “lamellar”, “tubular”, “mixed” and “transitional”, with representative tomographic slices (described in Section 2.2) shown in Figure 1. Mitochondria classified as “lamellar” (*Lam*) have cristae that appear in tomographic slices as pairs of straight or gently curved parallel lines spaced about 20 nm apart. The cristae are flat, sometimes branched, and generally extend across the organelle (with crista junctions on either end) and through the entire thickness of the reconstructed volume. The 2-D profiles of lamellar membranes contain frequent breaks that correspond to fenestrations (20–80 nm wide), described in detail in [35] (see also [38]). The mitochondria can be in an orthodox (low density, expanded matrix; Figure 1A) or condensed (dark, contracted matrix; Figure 1B) conformation [39]. “Tubular” (*Tub*) mitochondria have crista profiles that correspond to transverse (Figure 1C) and longitudinal (Figure 1D) views of irregularly packed, worm-like curved tubes, typically 50−90 nm in diameter. Since individual crista tubes wind in and out of the tomographic slabs, their connectivity at crista junctions was difficult to track and the occurrence of unattached tubular cristae could not be ruled out. In fact, small, unattached spherical vesicles (diameters 70−90 nm) are sometimes observed within tomograms of this class. “Transitional” (*Trans*) mitochondria have a distinctive 2-D signature, namely parallel rows of small (30−60 nm), circular and elongated membrane cross-sections (Figure 1E,F). In conventional micrographs, these might be mistaken for lamellar cristae but a tomographic analysis reveals that each row is composed primarily of tubes, narrower and shorter than those in the “tubular” class, interconnected with flat lamellar regions (see Section 3.4 and Section 3.5). This crista morphology was named “transitional” because it is similar to that observed in mitochondria of other cell types under conditions that induce lamellar-to-tubular crista transitions, as discussed in Section 4.2. “Mixed” (*Mix*) mitochondria contain roughly equal adjacent regions of two of the crista morphologies (Figure 1G,H). The overall impression is that the observed crista classes may represent a continuum and suggest a possible pathway for remodeling (see Section 4.2).

### 3.2. Correlations among Mitochondrial Size, Location and Crista Morphology

**Figure 2:**
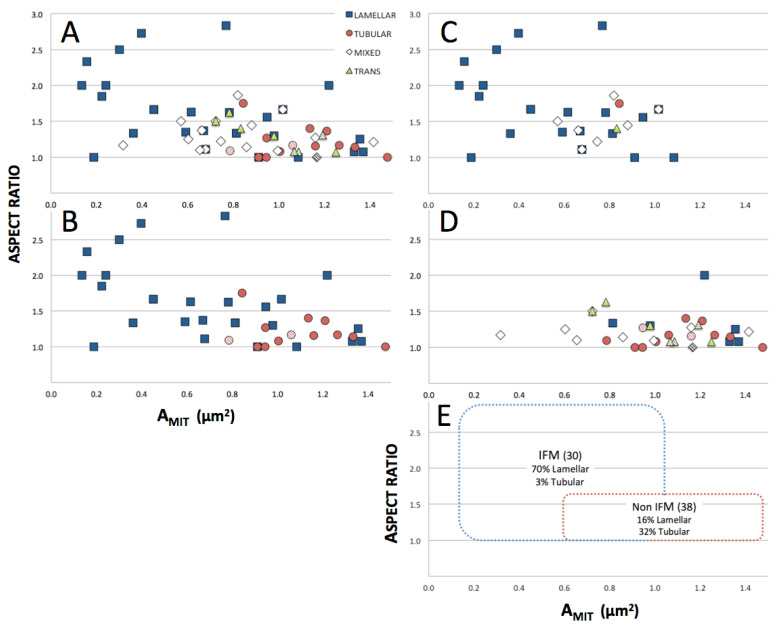
Correlations between aspect ratios and areas (A_MIT_) of mitochondrial cross sections in central tomographic slices. (**A**) All mitochondria. (**B**) Only mitochondria with lamellar and tubular crista morphologies. (**C**) Mitochondria located in interfibrillar regions (IFM). (**D**) Mitochondria not located in interfibrillar regions (NonIFM). (**E**) Rectangular boundaries enclosing all but one IFM and all but two NonIFM, with corresponding % of mitochondria with lamellar and tubular crista morphologies. Diagonally striped symbols correspond to mitochondria with a few locally swollen cristae (described in text).

The 68 mitochondria in the dataset, classified according to crista morphology, were further characterized in terms of basic structural parameters commonly applied to muscle mitochondria [40,41,42]. A plot of the aspect ratios of mitochondrial profiles in the central tomographic slices (AR = length of longest dimension/length of shortest dimension) vs. area of the cross sections (A_MIT_) is shown in Figure 2A for all mitochondria in the dataset, and for only *Lam* and *Tub* classes in Figure 2B. *Lam* mitochondria tend to fall to the left side (smaller areas) of the plot and are distributed over a wider range of aspect ratios than the other three classes. The converse is certainly true for *Tub* mitochondria, only one of which has an aspect ratio above 1.5 and none of which has an area smaller than 0.8 μm^2^.

**Figure 3:**
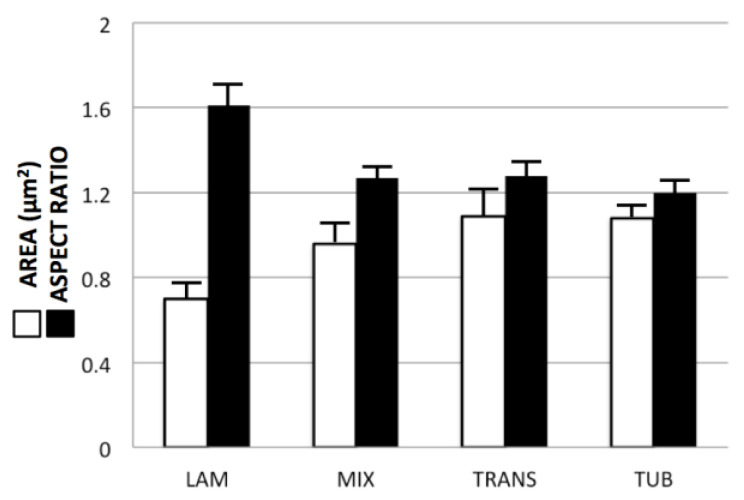
Chart of mean areas and aspect ratios (standard deviations indicated) for the cross-section profiles of all mitochondria in the dataset, classified according to crista morphology as described in the text. An unpaired T-test (GraphPad Prism 9) was used to compare values for *Lam* mitochondria vs. each of the other three crista morphology classes. Statistically significant two-tailed *p*-values were obtained for areas and aspect ratios with *Lam* vs. *Tub* (0.017, 0.009) and *Lam* vs. *Mix* (0.027, 0.017), but not *Lam* vs. *Trans* (0.108, 0.07) mitochondria.

Differences in the profile parameters (mean areas and aspect ratios) of the four morphological classes (Figure 2A,B) are quantified in the graphs of Figure 3. There is a progressive increase in mean A_MIT_ (from 0.70 to 1.08 μm^2^) and decrease in mean AR (from 1.61 to 1.20) as crista morphology varies from lamellar to mixed to transitional to tubular. In particular, differences between *Lam* and *Tub* mitochondria are highly significant (*p* < 0.02) for both areas and aspect ratios (see legend of Figure 3). Thus, ***mitochondria in the dataset with lamellar cristae tend to be smaller and more irregular in shape, while mitochondria with tubular cristae tend to be larger and rounder in profile.***

Table 1 summarizes the distribution of mitochondria of different crista morphologies in terms of location within the myocytes. For the purposes of this analysis, “interfibrillar” and “subsarcolemmal” classifications were restricted to include only mitochondria in close proximity (<50 nm) to myofibrils or to the plasma membrane, respectively. Mitochondria not meeting these criteria were classified as “other”, even if they were in clusters near myofibrils. The location classes were not evenly populated, with 30 interfibrillar, 29 other and only 9 clearly subsarcolemmal. Of the 30 interfibrillar mitochondria, 21 are *Lam* and another 7 *Mix* (lamellar/tubular). The 38 non-interfibrillar mitochondria in the dataset are roughly evenly distributed between mixed, transitional and tubular crista morphologies, although *Trans* mitochondria are absent from the subsarcolemmal region. The distributions of all classes of mitochondria in interfibrillar and non-interfibrillar regions on the AR vs. A_MIT_ plot are shown in Figure 2C and Figure 2D, respectively, and summarized for *Lam* and non-*Lam* mitochondria in Figure 2E. ***Clearly, mitochondria with lamellar crista morphology correlate with interfibrillar location in the dataset***.

### 3.3. Correlation between Crista Morphology and Membrane Density

**Figure 4:**
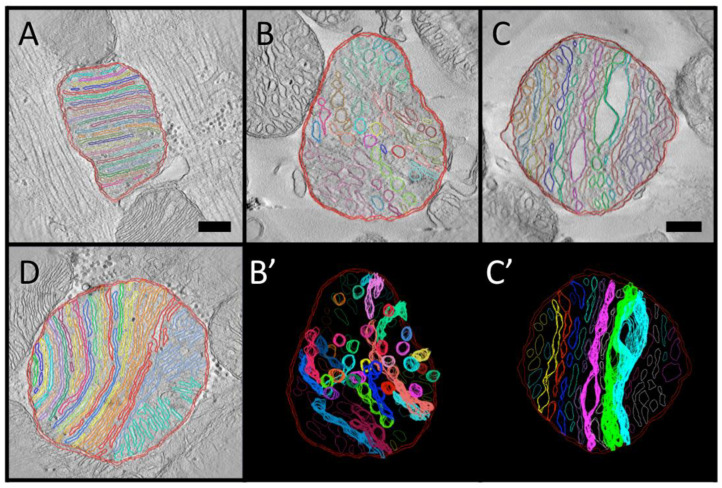
Examples of manually traced membranes in tomographic slices used to calculate crista membrane density. (**A**) An interfibrillar mitochondrion (IFM) with lamellar cristae. (**B**,**B’**) A non-interfibrillar mitochondrion (nonIFM) with tubular cristae. (**C**,**C’**) A nonIFM with transitional cristae and local swelling of three cristae. (**D**) The large nonIFM with lamellar cristae. Color coding of traces in (**A**,**C**,**C’**,**D**) indicates cristae that are interconnected immediately above or below the central slice shown. Color coding in (**B**,**B’**) is random since interconnectivity of tubular cristae is difficult to assess, as explained in text. Scale bars correspond to 250 nm. Scale bar in (**A**) also applies to (**B**,**B’**,**D**). Scale bar in (**C**) also applies to (**C’**).

As already noted, the evolution of mitochondrial cristae likely was driven by the selective advantage of packing more ATP-producing chemiosmotic membrane into the cell volume occupied by mitochondria. Thus, muscle cells (such as cardiac and flight muscle), which have a high and variable demand for ATP to power massive contractile machinery, tend to have mitochondria with the most densely packed cristae, e.g., [43]. Likewise, there is considerable evidence of physiological regulation of mitochondrial crista density, with increases correlated with endurance training in the human skeletal muscle [44], thyroid state in rat liver [45] and switching on oxidative metabolism in cancer cell lines [46], reviewed in [47]. Comparisons were made of the density of crista membrane packing for representative mitochondria with lamellar and tubular cristae, as well as for a large mitochondrion containing predominantly lamellar cristae with small subregions of other morphologies, and for a *Trans* mitochondrion with locally swollen cristae (the significance of which is explained below). Tomograms were selected for analysis in which the majority of membranes were normal to the viewing plane, which improved the reliability of manual tracing. Examples of traced images used for crista density measurements, along with partial 3-D renderings of a *Tub* and *Trans* mitochondrion, are shown in Figure 4.

The term “crista density” refers to the surface area of crista membranes contained within a mitochondrial volume (S_CRIS_/V_MIT_), measured in 2-D images as the length of crista membranes divided by the area of the mitochondrial cross section (L_CRIS_/A_MIT_). Metrics can vary, with “crista density” sometimes reported as the number of cristae per mitochondrial length, e.g., [42]. Also, many studies report the surface area of the entire inner membrane (S_IM_), cristae plus the inner boundary membrane (IBM), inside a mitochondrial volume (S_IM_/V_MIT_, measured in 2-D images as L_IM_/A_MIT_). Inner membrane density can vary considerably with cell and tissue type, with mean values for S_IM_/V_MIT_ of 21−24 μm^−1^ for rat liver mitochondria [45,48,49] and 37−41 μm^−1^ for rat and human heart mitochondria [50,51].

**Figure 5:**
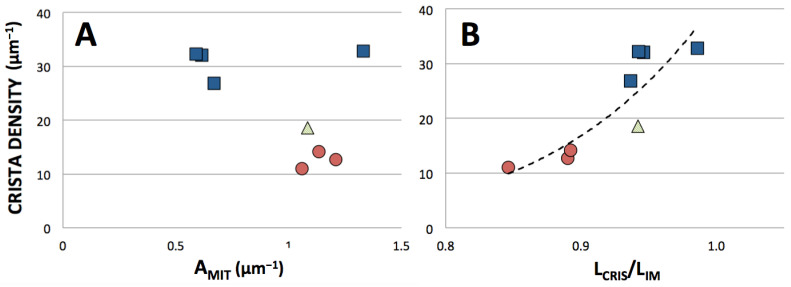
Dependence of crista density on the (**A**) mitochondrial cross-section area and (**B**) extent of inner membrane folding for the subset of cardiomyocyte mitochondria described in the text. Crista densities were measured for central tomographic slices as L_CRIS_/A_MIT_ where L_CRIS_ is the length of crista membranes contained within area A_MIT_, and extent of IM folding is the ratio of L_CRIS_ to L_IM_, the length of crista membranes plus IBM. Symbols are the same as in Figure 2.

Crista densities were compared for eight mitochondria in the tomographic dataset, seven with cross-section areas near the class mean value (3 *Lam*, 3 *Tub* and 1 *Trans*) and one *Lam* mitochondrion with about twice the mean class area. The three smaller *Lam* mitochondria are interfibrillar. The mean value of IM density (measured as L_IM_/A_MIT_) for this subset of mitochondria is 25.5 (±9.2) μm^−1^, below the reported range for rat heart mitochondria. However, the tomograms in the subset, as in the entire dataset, were not selected randomly for crista morphology. The mean crista density for the *Lam* mitochondria in the subset is 34.3 (±2.7) μm^−1^, near the reported range for rat muscle mitochondria, suggesting a variation in density of IM packing with crista morphology that has not been previously reported. This is illustrated in Figure 5A in terms of crista densities (measured as L_CRIS_/A_MIT_) plotted against mitochondrial area. The mitochondria with tubular cristae have the lowest crista densities, in the range 11−14 μm^−1^, while values for *Lam* mitochondria span 27−33 μm^−1^ over a range of mitochondrial areas that include those of the *Tub* mitochondria. The crista density of the *Trans* mitochondrion is ~19 μm^−1^, intermediate between the *Lam* and *Tub* ranges. Clearly, ***mitochondria with lamellar cristae have the densest crista membrane packing*** in the subset. This difference is not a trivial consequence of the crista geometries. The 3-D models of closely packed lamellar and tubular cristae (used for computational modeling of mitochondrial metabolism) have similar values for crista density (32−30 and 29−28 μm^−1^, respectively) across six-fold ranges in volume [13]. Thus, observed variations in crista membrane density associated with crista morphology in cardiac myocyte mitochondria reflect actual differences in membrane packing. This is confirmed by plotting crista density against L_CRIS_/L_IM_, a measure of the extent of IM infolding, for the mitochondria in the subset (Figure 5B). Increasing inner membrane folding clearly correlates with increasing crista density, as expected.

### 3.4. Local Three-Dimensional Analysis of Cristae in a “Transitional” Mitochondrion

**Figure 6:**
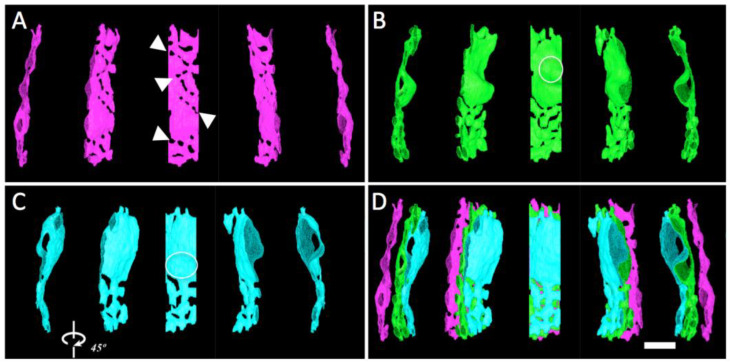
Surface models of three cristae from the *Trans* mitochondrion of Figure 4C,C’, displayed individually (**A**–**C**) and co-aligned as in the mitochondrion (**D**). Each view in the rows represents successive rotation by 45**°**. Arrows in (**A**) point to rows of fenestrations. Crista regions with flat, closely apposed membranes without fenestrations are circled in (**B**,**C**), as explained in text. Scale bar = 250 nm.

The complex morphology of cristae in “transitional” mitochondria was analyzed by manual tracing and surface rendering of select cristae in the *Trans* tomogram used for the crista density analysis (Figure 4C,C’). As evident in the 3-D models of Figure 6, there are two distinctly different zones in each crista: extended flat membrane regions that are either closely apposed (as in lamellar cristae) or separated due to crista swelling, and highly convoluted regions composed of short narrow tubes interconnecting wider flatter regions. Previously mentioned, crista fenestrations (20−80 nm openings in lamellar cristae that connect the matrix on either side) are evident in the crista of Figure 6A, but mostly absent from the flat, apposed crista membrane regions in the other two cristae (circled in Figure 6B,C). Interestingly, the fenestrations in the crista of Figure 6A appear to align in rows (arrows) that parallel the separations between tubular regions in the cristae of Figure 6B,C. This raises the possibility that the lateral merger of fenestrations, possibly triggered (in this case) by crista swelling, could create the convoluted, interconnected tubular membrane regions that are the signature of “transitional” mitochondria (see Section 4).

### 3.5. Complete Three-Dimensional Analysis of Cristae in a Large “Lamellar” Mitochondrion

**Figure 7:**
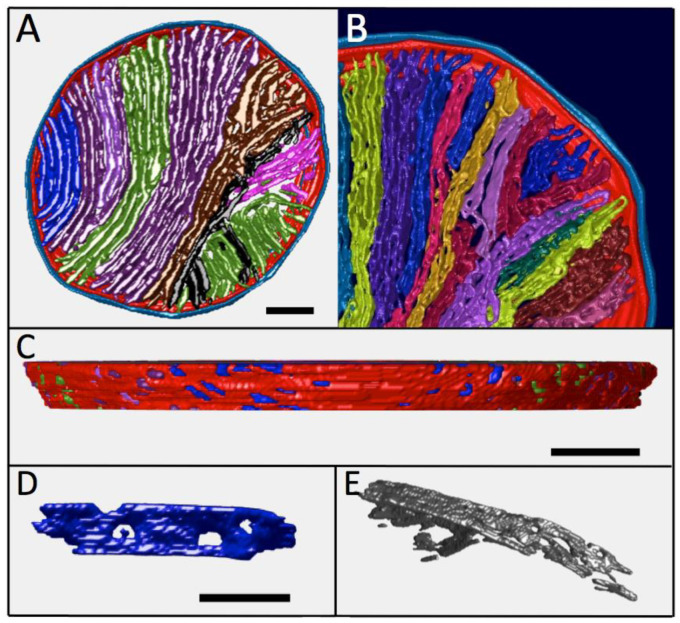
Surface models of the *Lam* mitochondrion of Figure 4D. (**A**) Top view of the tomogram with individual cristae sealed at top and bottom (for visual simplicity). Cristae are color coded; left to right: blue #1–4, purple #5–10, green #11, purple #12, brown #13, black #14, green #15, pink #16. (**B**) Top view of the upper-right quadrant, with open (unsealed) cristae. 2X zoom and colors changed to better discern crista branches and junctions with the IBM. (**C**) Side view of the mitochondrion (after 90° clockwise rotation) revealing the openings in the IBM corresponding to crista junctions (CJs), several of which are slots in this region. (**D**) Side view of a typical lamellar crista (#3) showing fenestrations. (**E**) Oblique view of the “transitional” crista (#14), which has a length of 1.1 μm. Scale bars = 200 nm. The scale bar in (**C**) also applies to (**B**).

The large, densely packed *Lam* mitochondrion of Figure 4D was used in the development of a novel protocol for the 3-D analysis of complex membranes inside tomograms using open source tools [35]. Although not yet automated, the protocol provides a facile new approach to render and extract functionally important structural parameters from tomographic data sets. Top-down and side views of the membrane surfaces in the *Lam* mitochondrion of Figure 4D are presented in Figure 7, along with several dissected individual cristae. The mitochondrion displays an obvious gradation in crista topology from left to right in the top view, changing from lamellar with no or one branch (cristae #1−10) to extensively branched lamellar (#11−13), transitional (#14) and tubular (#15, 16) morphologies. The high degree of branching occurs near the middle of the mitochondrion and results in the efficient space-filling of the circular cross-section. This maintains a high crista membrane density in the lamellar region, 55 μm^−1^ for cristae #1–10 and 65 μm^−1^ for cristae #11–13. By contrast, crista density decreases to 47 μm^−1^ in the subregion (cristae #14–16) containing cristae with more tubular shapes. Note that the mean density of crista membranes in this mitochondrion (58 μm^−1^) is approximately 60% higher than that estimated from 2-D measurements (Figure 5). There are two apparent reasons for the discrepancy: (i) membrane surface areas were measured for the model in Figure 7A in which cristae are sealed on either side of the tomogram, which adds extra membrane surface; (ii) this surface rendering procedure more closely tracks the texture and complexity of membranes than manual tracing, which can also miss highly curved surfaces, e.g., [48]. The process of parameter optimization (spatial filtering, thresholds, mesh sizes) relative to the desired level of detail and expected resolution in these 3-D volumes is ongoing.

**Figure 8:**
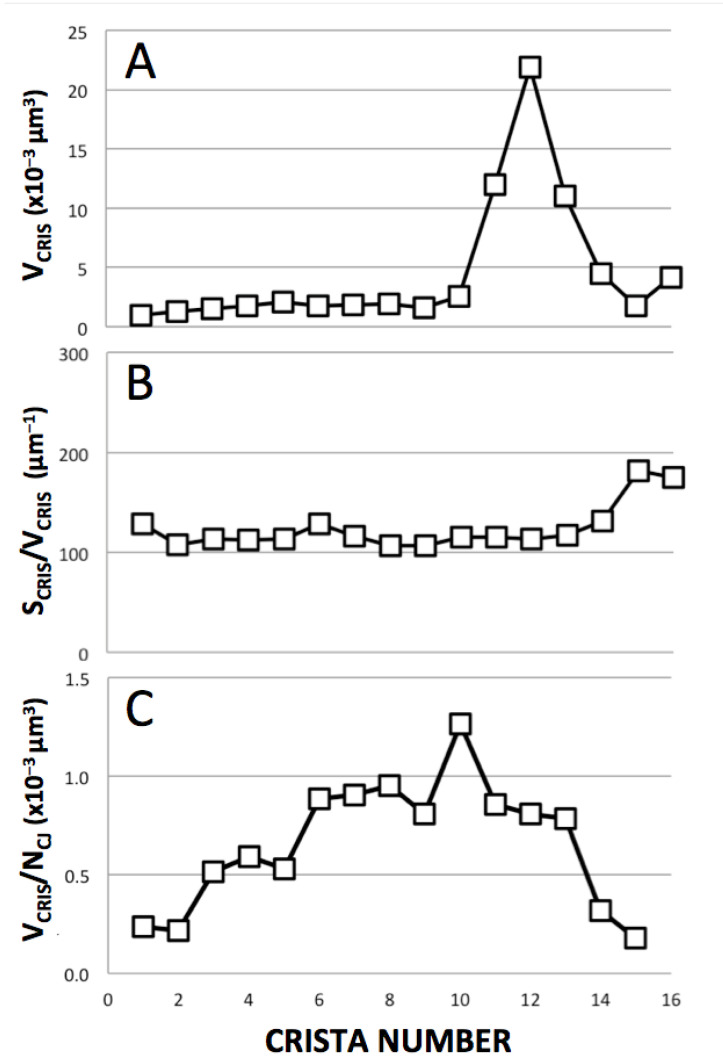
Structural parameters for individual cristae in the *Lam* mitochondrion of Figure 7. Cristae are numbered from left to right as in Figure 7A. (**A**) Crista volumes, V_CRIS_. The large increases in V_CRIS_ correspond to extensive branching near the middle of the mitochondrion. (**B**) Surface-to-volume ratios, S_CRIS_/V_CRIS_. (**C**) Volume per crista junction opening, V_CRIS_/N_CJ_.

Plots of structural parameters for the “sealed” cristae in Figure 7A are shown in Figure 8. Despite an order of magnitude increase in crista volume (V_CRIS_) associated with extensive branching (cristae #11–14), the surface-to-volume ratios of cristae (S_CRIS_/V_CRIS_), important for processes that depend on facilitated membrane transport, remain constant throughout the lamellar region, ~110 μm^−1^, and increases to ~180 μm^−1^ for the crista region with more tubular morphology (consistent with simple geometric considerations; Section 4.1.1). Numerous crista junctions (CJ) are visible in the side view of the mitochondrion (Figure 7B) as circular or slot-like openings in the inner boundary membrane, with dimensions that vary considerably. Round CJ openings fall in the 15–40 nm range while slot openings can be up to 70 nm in length, generally consistent with previous descriptions [5,8,52]. The ratio of the volume of individual cristae to the number of CJ openings (V_CRIS_/N_CJ_), which impacts processes that are rate-limited by lateral diffusion in and out of cristae, plateaus at around 1 × 10^−3^ μm^3^. The lamellar cristae in this mitochondrion contain numerous fenestrations, readily visible in Figure 7D,E, that connect the matrix “layers” on either side of the crista “barriers”. The fenestrations vary in width from 20−80 nm (mean = 36 nm) and are randomly arranged on most cristae in this *Lam* mitochondrion, e.g., Figure 7D; see also [38]. However, there is a suggestion of crowding and the linear alignment of fenestrations in the “transitional” crista (#14, Figure 7E), similar to that seen in the *Trans* mitochondrion (Figure 6A). The functional significance of fenestrations aside from facilitating “matrix mixing” across cristae is discussed in Section 4.2.

## 4. Discussion

### 4.1. Bioenergetic Rationale for Location of “Lamellar” Mitochondria in the Interfibrillar Space

This survey of mitochondria in rat cardiomyocytes utilizes information in a 3-D dataset provided by transmission electron tomography. Although relatively small compared to 3-D datasets provided by lower-resolution “volume EM” techniques like serial block-face SEM (e.g., [53]), significant correlations were found among organelle shape, cell location and morphology, and density of crista membranes. Zooming in on cristae within individual mitochondria revealed local variations in membrane topology and packing that may relate to crista dynamics and remodeling (discussed in Section 4.2).

The high aspect ratios and irregular profiles of the interfibrillar mitochondria in the dataset (“longitudinal” views) are consistent with previous TEM studies of myocytes, e.g., [40,41]. “Transverse” views in published micrographs and tomograms [23,54] indicate that interfibrillar mitochondria are closely packed, interconnected cylinders, with frequent straight interfaces between adjacent organelles, e.g., Figure 1A,B; also Figure 1C, a mitochondrial cluster near myofibrils. Away from the crowded interfibrillar zones, mitochondrial profiles tend to be rounder and wider, consistent with less elongated, more spherical/ovular 3-D shapes. The novelty in the current analysis is the apparent bias for mitochondria with lamellar cristae, which predominate in the regions between myofibrils, to have higher crista membrane densities than mitochondria with tubular cristae. This raises an obvious question whether there is an advantage, energetic or otherwise, for positioning mitochondria with lamellar cristae in the interfibrillar space.

#### 4.1.1. Influence of Crista Morphology on ATP Output

The nano-scale compartmentalization of mitochondria creates barriers to the internal diffusion of solutes that can impact function. Examples include the incomplete release of cytochrome c from cristae during apoptosis, and the formation of pH and electrical gradients within and between cristae [8,9,11]. Computational studies suggest restricted internal diffusion of metabolites can affect both the rate of synthesis of ATP and its export to the cytoplasm [12,13]. In the case of cardiac muscle mitochondria, where ATP production is carefully regulated to meet high and variable energy demands (e.g., [29,55]), crista would be expected to adopt shapes that minimize the adverse effects of diffusion on ATP production. The above findings from electron tomography suggest that this is indeed the situation in cardiomyocytes.

**Figure 9:**
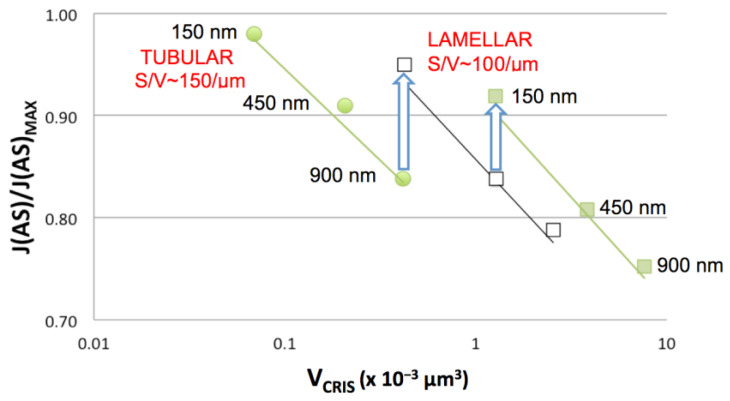
Dependence of flux of ATP Synthase, J(AS), on shape, length and volume (V_CRIS_) of cristae. Data is re-plotted from published computer simulations that used idealized 3-D spatial models of mitochondria and a reduced mathematical model for ATP production [13]. Physiological conditions were used for which J(AS) varies directly with the matrix [ADP] (cytosolic [ADP] set to 37 μM). Spatial models: circles, 4 × 4 parallel array of 20-nm-wide tubes spaced 20 nm apart; squares, four parallel lamellar compartments, walls spaced 20 nm apart with widths of 150 nm (open symbols) or 450 nm (closed symbols). Crista lengths are indicated in black and S/V ratios in red. The reference J(AS)_MAX_ (flux without crista compartments) is the same for all three curves. Vertical blue arrows connect data points for different crista types of equivalent volumes.

In general, the rates of processes that involve the facilitated membrane transport of reactants increase with the surface-to-volume (S/V) ratio of the compartments [56]. Tubular shapes tend to have larger S/V ratios than extended flat compartments, e.g., S/V -> 4/D for long right-circular cylinders with diameter D, while S/V -> 2/D for wide flat plates separated by D. Consistent with this expectation, crista S/V ratios inside the mitochondrion of Figure 7 are greater by almost a factor of two for partly tubular cristae relative to lamellar cristae (180 μm^−1^ vs. 110 μm^−1^, Figure 8B). The higher S/V ratios should confer an advantage on tubular cristae when ADP is limiting, i.e., the matrix [ADP] regulates the rate of ATP synthesis [13]. Under these physiological conditions, steady-state ATP production is tuned to the rate of transport of ADP across the inner membrane via the adenine nucleotide translocase. Figure 9 is a re-plot of data from computer simulations in [13], which employ a model for mitochondrial ATP production at a constant inner membrane potential, based on rate equations in [57]. (Spatial models and parameters used are described in the figure legend). Under these conditions, the flux of ATP synthase, J(AS), is predicted to be 25% higher for short tubular cristae compared to large lamellar cristae. However, when comparing cristae of equivalent lengths (relevant to lateral diffusion of metabolites in and out of the cristae), the advantage of the tubular shape for J(AS) decreases to 10% or less. Moreover, when considering equivalent crista volumes, the shape advantage reverses, with wide lamellar cristae supporting larger fluxes of ATP synthase than narrow tubes (blue arrows in Figure 9). Thus, remodeling the IM from tubular to increasingly wide lamellar cristae as mitochondrial size increases would appear to be a bioenergetically optimal strategy.

Lamellar cristae tend to predominate in the interfibrillar region of striated muscle, where optimizing ATP production is a priority [24]. Since the dimensions of mitochondria spanned by cristae in our dataset typically range from 0.5 μm (Figure 4A) to over 1.0 μm (Figure 7), lamellar cristae would support equivalent or faster ATP synthase fluxes than longer crista tubes. The two-fold greater density of crista membranes in *Lam* as compared to *Tub* mitochondria would ensure that the former crank out considerably more ATP per unit volume occupied, since ATP output is the product of flux and membrane surface area, J(AS) × S_CRIS_.

Of course, there also may be other considerations besides internal diffusion of metabolites that favor lamellar crista morphology. One relates to the physical organization of the respiratory chain, in particular, ordered supercomplexes of respiratory complexes I-III-IV (RSCs) that have been shown to increase efficiency of oxidative phosphorylation [58,59,60]. There is evidence that the assembly of the large, planar supercomplexes depends on crista shape, since the remodeling of cristae in mouse fibroblasts, from flat (“tight”) to swollen by ablation of OPA-1, correlates with decreased RSC formation and respiratory control ratios [59]. Such a requirement for RSC assembly would provide an additional bioenergetic advantage to *Lam* mitochondria.

An apparent disadvantage of wide lamellar cristae from the perspective of ATP output is the paucity of highly curved membrane regions at which ATP synthase dimers normally reside [61,62]. Most lamellar crista membranes in the dataset extend through the entire thickness of the tomograms (e.g., Figure 7B), suggesting that they are several hundred nanometers wide. In this case, the only regions suitably curved for ATP synthase dimers are the crista rims that run parallel to the IBM between crista junctions. The solution to this conundrum may be the numerous fenestrations that occur in the cristae, which have a curvature direction and radius of curvature equivalent to that of crista folds. An important function of the fenestrations may be to serve as a repository for ATP synthase dimers, increasing their frequency and evenly distributing them across otherwise flat crista surfaces. This scenario might improve the efficiency of ATP production, e.g., by a hypothesized “proton trap” mechanism [61] and/or by reducing lateral metabolite gradients in the matrix layers between cristae. Fenestrations stabilized by ATP synthase dimers might also increase the mechanical stability of lamellar cristae and resist crista swelling due to osmotic fluctuations [2]. Since ATP synthase dimers induce curvature in liposomes, it seems likely that they participate in the formation of fenestrations in lamellar cristae, certainly a testable hypothesis.

#### 4.1.2. Crista Responses to Physical Stresses

A recent review on the “energy metabolism design” of the striated muscle draws attention to a mostly overlooked aspect of myocyte mitochondria, namely, their exposure to physical stresses in the region between myofibrils [25]. These include shear forces, exacerbated by the attachment of mitochondria to fibers in the sarcomeres, in both longitudinal (stretching) and transverse (compression) directions during the contraction cycle. The reshaping of interfibrillar mitochondria associated with the stretching and contracture of rabbit cardiac muscle was inferred in a recent electron tomographic study [54]. The slow dynamics and turnover of mitochondria in cardiac muscle [23,63] suggest individual organelles may undergo large numbers (10^5^–10^6^) of deformation cycles due to these physical stresses. The lamellar cristae of cardiomycyte mitochondria are extended, closely packed parallel plates, with numerous junctions or connections (every 100 nm or so) to the IBM (e.g., Figure 7C), which itself has numerous connections via MICOS components to the outer membrane [64]. This basic design is roughly analogous to “shear walls”, architectural elements implemented to survive lateral stresses from seismic events [65]. By contrast, tubular cristae are long, flexible, generally disorganized structures that connect at single junctions to the peripheral IBM. It may well be that lamellar cristae are selected for the IFM because they are more resilient than tubules, better able to reversibly deform in response to physical stresses inherent to the interfibrillar space.

### 4.2. Mechanisms of Crista Remodeling

Lamellar and tubular cristae typically co-exist in metazoan mitochondria, with crista remodeling an ongoing process [20,66]. As already noted, IM topology is regulated by membrane-shaping proteins and protein complexes, including MICOS, ATP synthase oligomers and OPA-1, their mutual interactions and their interactions with other proteins and lipids such as MICU1 and cardiolipin (see also [17,67,68,69,70]). Changes in crista morphology are associated with cell development, aging and disease, and can be caused by mutations in or the processing of membrane-shaping proteins, as well as changes in lipid composition [42,64,71,72,73]. There is evidence that cristae originate as tubes extending from the IBM at the junctions formed by MICOS, and grow by fusion into larger compartments, which in turn can flatten to form lamellar crista, with sharp bends in the IM induced and/or stabilized by dimer ribbons of ATP synthase (as noted above) [20,74]. In this scenario, crista growth by fusion could be favored by conditions that increase inter-crista contact, such as a reduction in volume of the mitochondrial matrix [2,75]. In the case of cardiomyocytes, crowding of mitochondria in the interfibrillar space is likely associated with compressive forces that would decrease the mitochondrial volume [76], thereby favoring crista contact and fusion. This is consistent with the observation that interfibrillar mitochondria (IFM) tend to have smaller, more irregular profiles and flat interfaces with neighbors. Conversely, the lower crista density of *Tub* non-IFM compared to *Lam* IFM (Section 3.3) arises primarily from the former’s 80% larger mean cross-section area (1.14 vs. 0.63 μm^2^). Thus, cardiomyocyte mitochondria not situated in the rows between myofibrils may not be subject to the same physical constraints on shape and volume as IFM. In fact, there is compelling evidence that the mitochondria in cardiomyocytes are a single, continuous population [28,40], suggesting that differences in shape and IM topology might reflect their local environment, including compressive and osmotic forces (see also [77]).

The “transitional” crista class was so-named because of its similarity to morphologies previously observed in model systems undergoing transition from lamellar to tubular crista morphology. The closest examples occur in *Drosophila* mitochondria in (i) neuronal cells bearing a disease-causing mtDNA mutation in *ATP6* [78] and (ii) the flight muscle after exposure to oxygen stress or bearing the “swirl” mutation [79,80]. In these systems, tubular cristae appear as extensions from lamellar regions, similar to crista #14 in the mitochondrion of Figure 7, which is located between a branched lamellar crista and two tubular cristae. The cristae in the *Trans* mitochondrion of Figure 6 suggest successive steps in a pathway for forming tubular extensions: (1) lateral diffusion of pore-like fenestrations away from a region of local swelling of lamellar cristae, (2) alignment of the fenestrations in rows and (3) fusion of the rows into linear “crevices” that define individual tubes in the crista plane. This model is consistent with the above suggestion that fenestrations are lined with ATP synthase dimers which, when the fenestrations fuse, might re-assemble into spiral ribbons that wrap around (or induce the formation of) tubular cristae [81]. Fenestrations may be needed to supply ATP synthase dimers during remodeling only for large lamellar cristae, in which crista rims are remote. For smaller mitochondria, which do not normally have crista fenestrations, ATP synthase dimers released from nearby crista rims by swelling might be sufficient to initiate tubule formation. Whether the transient formation of fenestrations is an intermediate step in such cases is another testable hypothesis.

The driving force for crista remodeling by the above mechanism is whatever triggers crista swelling. This could include an osmotic imbalance (e.g., due to changes in ion transport) that induces the rapid movement of water from the matrix to the intracristal space when crista junctions are “closed” [2,11]. Another candidate is excess mitochondrial ROS production, associated with mitochondrial dysfunction and hypothesized to decrease with crista widening [82]. Oxidative stress is implicated in the transitions from lamellar to tubular cristae in the two *Drosophila* examples above (see [83]), as well as those observed with P450 induction in Leydig cells [84] and the onset of ALS in motor neurons of a SOD1 mouse model [85]. Potential ROS targets involved in crista swelling (or tightening [86]) include the aforementioned set of crista-shaping proteins and cardiolipin, although the actual mechanism is not yet defined.

## 5. Conclusions

The cell expends considerable resources on regulating mitochondrial inner membrane topology, consistent with its central role in energy metabolism. This is especially true of cardiac muscle, where peak ATP production and structural resilience are priorities. The current structural analysis provides novel, significant evidence (despite the limited size of the tomographic dataset) that crista morphology and membrane density are optimized for bioenergetic performance in mitochondria immediately adjacent to myofibrils. Moreover, zooming inside individual mitochondria reveals gradations in membrane topology not previously reported that suggest a novel crista-remodeling pathway involving fenestrations common to lamellar cristae in myocyte mitochondria. Given the central role of cristae in energy metabolism, and the fact that structural aberrations are associated with numerous diseases, understanding the factors that regulate inner membrane topology is fundamentally important. Clearly, further work is needed to shore up these novel observations and provide additional structural clues to help define the triggers and molecular players involved in the transitioning of cristae between tubular and lamellar shapes. Electron tomography (eventually of cryogenically prepared tissue [87]) will continue to be an important tool for achieving this goal.

## Figures and Tables

**Table 1:** Distribution of mitochondria with different crista morphologies according to location within cardiac myocytes *.

Morphology	Interfib	Subsarc	Other	Total	Swollen
Lam	21	1	5	27	0
Mix	7	4	8	19	1
Trans	1	0	8	9	3
Tub	1	4	8	13	2
Total	30	9	29	68	6

* Data from Figure 2C,D with crista morphologies and cell locations defined as described in text, as are abbreviations for crista morphologies. Interfib = Interfibrillar; Subsarc = Subsarcolemmal. Also shown is the distribution of mitochondria with locally swollen cristae (diagonally striped symbols in Figure 2).

## Data Availability

Electron tomograms used in this study will be made available upon reasonable request. We intend to deposit the data in an appropriate 3D-EM databank in the near future.
